# Test on existence of histology subtype-specific prognostic signatures among early stage lung adenocarcinoma and squamous cell carcinoma patients using a Cox-model based filter

**DOI:** 10.1186/s13062-015-0051-z

**Published:** 2015-04-07

**Authors:** Suyan Tian, Chi Wang, Ming-Wen An

**Affiliations:** Division of Clinical Epidemiology, First Hospital of Jilin University, 71Xinmin Street, Changchun, Jilin 130021 China; Department of Biostatistics and Markey Cancer Center, University of Kentucky, 800 Rose St., Lexington, KY 40536 USA; Department of Mathematics, Vassar College, Poughkeepsie, NY 12604 USA

**Keywords:** Non-small cell lung cancer (NSCLC), Adenocarcinoma (AC), Squamous cell carcinoma (SCC), Cox model, Prognosis, Histology-subtype specific, Gene expression barcode, Feature selection algorithm

## Abstract

**Background:**

Non-small cell lung cancer (NSCLC) is the predominant histological type of lung cancer, accounting for up to 85% of cases. Disease stage is commonly used to determine adjuvant treatment eligibility of NSCLC patients, however, it is an imprecise predictor of the prognosis of an individual patient. Currently, many researchers resort to microarray technology for identifying relevant genetic prognostic markers, with particular attention on trimming or extending a Cox regression model.

Adenocarcinoma (AC) and squamous cell carcinoma (SCC) are two major histology subtypes of NSCLC. It has been demonstrated that fundamental differences exist in their underlying mechanisms, which motivated us to postulate the existence of specific genes related to the prognosis of each histology subtype.

**Results:**

In this article, we propose a simple filter feature selection algorithm with a Cox regression model as the base. Applying this method to real-world microarray data identifies a histology-specific prognostic gene signature. Furthermore, the resulting 32-gene (32/12 for AC/SCC) prognostic signature for early-stage AC and SCC samples has superior predictive ability relative to two relevant prognostic signatures, and has comparable performance with signatures obtained by applying two state-of-the art algorithms separately to AC and SCC samples.

**Conclusions:**

Our proposal is conceptually simple, and straightforward to implement. Furthermore, it can be easily adapted and applied to a range of other research settings.

**Reviewers:**

This article was reviewed by Leonid Hanin (nominated by Dr. Lev Klebanov), Limsoon Wong and Jun Yu.

**Electronic supplementary material:**

The online version of this article (doi:10.1186/s13062-015-0051-z) contains supplementary material, which is available to authorized users.

## Background

Non-small cell lung cancer (NSCLC) is the predominant histological type of lung cancer, accounting for up to 85% of cases [1]. The overall five-year survival rate of NSCLC is estimated extremely low at roughly 15% due to late discovery of disease among more than two-thirds of NSCLC patients, for whom surgical resection is no longer an option [2]. Moreover, even among early stage patients who have surgery, roughly 50% die of tumor recurrence [3]. Clinical studies [4,5] have demonstrated that adjuvant chemotherapy significantly improves the survival of NSCLC patients at early stages. Disease stage is commonly used to determine adjuvant treatment eligibility, however, it is quite possible that a proportion of stage I patients have poorer prognosis and may benefit significantly from adjuvant chemotherapy while some relatively good prognosis stage II patients may not benefit significantly from adjuvant chemotherapies. Therefore, identification of poor prognosis of early stage NSCLC patients will assist in the prescription and administration of additional therapeutic interventions, which might potentially lead to better survival for the patients with poor prognosis.

Microarray technology allows simultaneous monitoring of thousands of genes and measuring of their expression values. A major challenge in the analysis of data from microarray experiments is high dimensionality, i.e. the number of genes is much larger than the sample size. However, with a feature selection algorithm, the original set of genes can be reduced to a small gene subset that is informative of the underlying differences among phenotypes. A feature selection algorithm can be classified into one of three categories – filter, embedded and wrapper – depending on how the model fitting is combined with the subset selection [6]. Details of these categories, including relative merits and examples, are provided in a review by Saeys et al. [6].

Many of the current approaches for identifying relevant genes associated with survival phenotypes using microarray data focus on trimming or extending a Cox regression model [7], a commonly used model of analyzing survival data in traditional clinical settings. For instance, Gui and Li [8] proposed a novel feature selection algorithm called *LARS-cox*, which uses the least angle regressions (LARS) algorithm to obtain the solutions to a Cox model with an L1 penalty. In an L1 penalty model, also referred to as the least absolute shrinkage and selection operator (Lasso) model, the objective function to be minimized is the negative log likelihood function plus the sum of the absolute value of the coefficients, where the sum of the absolute value of the coefficients is restricted to be less than some constant *s*. Subsequently, Sohn et al. [9] applied the gradient lasso algorithm to a Cox model with an L1 penalty instead and named the proposed method *glcoxph.* Compared to *LARS-cox*, *glcoxph* claims to have prefect stability, save computing time, and be more likely to achieve the global optimum [9].

Adenocarcinoma (AC) and squamous cell carcinoma (SCC), each approximately accounting for 40% of NSCLC cases, are two major histology subtypes of NSCLC. Fundamental differences have been found between the two subtypes in the underlying mechanisms of tumor development, growth, and invasion [10,11]. Therefore, successful classification of NSCLC patients into their corresponding subtypes is of clinical importance. Many efforts [11-15] have been devoted to identifying subtype-specific genes, aiming at a precise diagnosis of NSCLC subtype and a feasible guide for personalized medicine. Many of those studies proposed and adopted a novel feature selection algorithm. The fundamental differences between AC and SCC of NSCLC patients motivated us to speculate that specific genes are related to survival rates for each histology subtype. To the best of our knowledge, however, all proposed Cox-model extensions ignore the histology subtype information. Their primary objective is to discriminate patients into subgroups with different survival profiles based on gene expression data, that is, selection of relevant gene subsets associated with prognosis for the whole study population regardless of specific subpopulation characteristics.

In this article, we propose a simple feature selection algorithm using a Cox regression model as the filter to evaluate genes individually for potential subtype-specific prognostic genes. Additionally, we explore the use of expression barcode values [16,17], in which a gene is deemed as either expressed or silenced based on its actual expression values. The expression barcode algorithm can detect a gene with nonlinear association to the outcome. The novel features of the proposed method are that it aims specifically at identifying subtype-specific prognostic genes plus it is conceptually simple and straightforward to implement.

## Methods and materials

### Experimental data

The lung cancer microarray experiment was conducted by [18] to assess the appropriation and accuracy of their previously identified 15-gene prognostic signature from another independent NSCLC microarray experiment [19].

The data were deposited into the Gene Expression Omnibus (GEO) repository under accession number GSE50081. It was hybridized on Affymetrix HGU133 Plus 2.0 chips. In this cohort, there were 181 early-stage NSCLC patients who did not receive any adjuvant therapy. Because we were only interested in AC and SCC subtypes, we excluded those samples with ambiguous histologic subtype labels and those other than AC and SCC, resulting in 127 AC and 42 SCC samples.

### Pre-processing procedures

Raw Affymetrix data (CEL files) were downloaded from the GEO repository and expression values were obtained using the *GCRMA* [20] algorithm. Data normalization across samples was carried out using quantile normalization and the resulting expression values were log_2_ transformed.

First, only probe sets that demonstrated a certain degree of variation across samples were selected. Specifically, probe sets with standard deviation (SD) below 0.1 were regarded as non-informative and eliminated. Then moderated t-tests using limma [21] were conducted to identify the differentially expressed genes (DEGs) between SCC and AC. Exclusion of those non-DEGs was the second stage of the filtering, and the cutoff for the false discovery rate (FDR) was set at 0.05. There were 5,465 down- and 5,484 up-regulated probe sets, corresponding to 6,202 unique DEGs. To deal with multiple probe sets matched to one specific gene, the one with the largest fold change was kept.

When using the barcoded values, the probe sets that expressed at extremely high (>95% in AC and >90% in SCC) or low frequencies (<5% in AC and <10% in SCC) were eliminated. This additional filtering was necessary to avoid problems associated with complete separation **–** where one specific gene is expressed in all samples of one subtype and silenced in the other. We used different cutoffs for AC and SCC mainly because the number of SCC samples is only one-third that of AC samples. The resulting 2,207 probe sets corresponding to 1,889 unique DEGs were fed into the downstream analysis.

### Methods

#### The Cox proportional model to identify subtype-survival relevant genes

**Figure 1:**
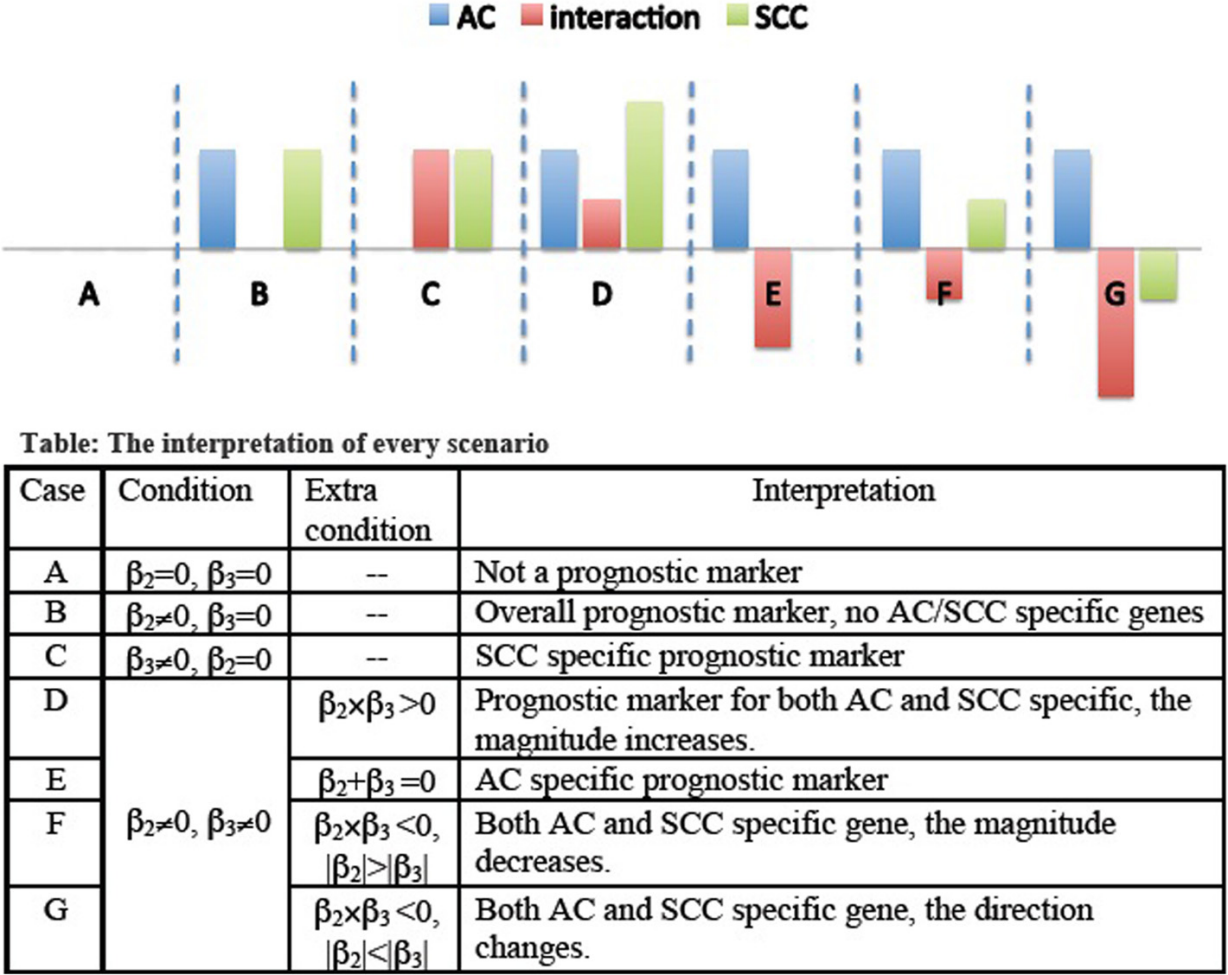
**Scenarios of the Cox-model filter and their interpretation.** β_2_ and β3 are the coefficients for the gene term and gene × subtype interaction term in Equation 1, and they are the parameters of interest. Ideally, non-overlap of genes fitting case B and those fitting case E suggests existence of mutually exclusive subtype-specific prognostic genes. Character **A-G** represent different situations (or cases) for the proposed model.

To identify genes informative of survival rate for AC/SCC histology subtypes, a Cox model was fit on each gene. Specifically, for patient *i* (*i =* 1,…,N_j_) of subtype *j* (*j =* 0,1 representing AC and SCC, respectively), t_ij_, δ_ij_, X_ij1_,…,X_ijp_ are observed. Here, δ_ij_ is the censoring indicator equalling 1 if this patient is dead and 0 otherwise, t_ij_ denotes survival time if δ_ij_ = 1 and censoring time otherwise, and X_ij_ = (X_ij1_,…,X_ijp_)^T^ represent actual expression values or barcoded expression values for the p genes under consideration. Then the hazard function of patient *i* for gene *g* (*g =* 1,…,p) is given by,

1 $$ {\lambda}_{ijg}(t)={\lambda}_{0g}(t) exp\left({\beta}_{1g}I\left(j=1\right)+{\beta}_{2g}{X}_{ijg}+{\beta}_{3g}I\left(j=1\right)\times {X}_{ijg}\right) $$

where λ_0g_(t) is an unknown baseline hazard function, and I(j = 1) is an indicator for the histology subtype of patient *i* belonging to SCC. Here β_2g_ and β_3g_ are the parameters of interest, with β_2g_ representing the change in log hazard rate associated with 1-unit increase in the actual expression value of gene *g* among AC and β_3g_ representing the additional change in log hazard rate associated with the SCC subtype. Of particular interest is when there is no overlap between subtype-specific prognostic genes. These genes can be identified as those that have either β_2g_ or β_2g_ + β_3g_, but not both, being statistically significantly different from zero. Figure 1 illustrates all possible scenarios.

It is worth mentioning that both λ_0g_ and β_1g_ are gene-specific, meaning the subscript *g* in these two terms cannot be dropped. Here, λ_0g_ takes into account the influence of relevant genes other than gene *g* and clinical covariates on the hazard rate. β_1g_ may assess the imbalance among those factors in AC versus SCC subtypes; its theoretical value is zero. We used the Benjamini and Hochberg procedure [22] to correct for multiple comparisons.

#### Barcode algorithm

In the barcode algorithm by McCall et al. [17], the expressed genes are coded with 1’s and the silenced genes are coded with 0’s. Briefly, McCall et al. [17] used a mixture model to fit the silenced and expressed distribution of observed log_2_ transformed intensity values for each gene. The mixture model is as follows,

$$ \begin{array}{l}{y}_{ig}\Big|{\mu}_g\sim \left(1-{p}_g\right)\times N\left({\mu}_{g,}{\tau}_g^2\right)+{p}_g\times U\left({\mu}_g,{S}_g\right)\\ {}{\mu}_g\sim N\left(\xi, {\lambda}^2\right)\\ {}{\tau}_g^2\sim IG\left(\alpha, \beta \right)\end{array} $$

where y_ig_ is the observed log_2_ intensity for gene *g* in sample *i*, and is assumed to follow a normal distribution of N(μ_g_, τ_g_^2^ ) if the *g*th gene is silenced or a uniform distribution of U(μ_g_, S_g_) if the *g*th gene is expressed. Here, μ_g_ denotes the mean of silenced genes and S_g_ denotes the saturation value. Then, silenced means and variances for each gene are assumed to follow normal and inverse gamma distributions, respectively. By introducing a hierarchical model structure, and in particular the higher-level parameters, i.e., α, β, ξ, and λ, more stable estimates of τ_g_^2^ can be obtained because information can be borrowed and shared across genes, leading to a shrinkage of individual estimates toward the overall average.

To determine whether a gene is more likely to be silenced or expressed, the standardized intensity value (*y*_*ig*_ − *μ*_*g*_)/*τ*_*g*_ was calculated. Using a fixed threshold value C, the expression barcode for a gene, a vector of 1’s and 0’s (indicating expressed and silenced) is defined as,

$$ {b}_{ig}=\left\{\begin{array}{cc}\hfill 1\hfill & \hfill \Phi \left(-\left({y}_{ig}-{\mu}_g\right)/{\tau}_g\right)<C\hfill \\ {}\hfill 0\hfill & \hfill otherwise\hfill \end{array}\right. $$

where Φ is the cumulative density function of a standard normal. Parameter estimation in this hierarchical model is done using a modified EM algorithm; details are available in the supplementary material of [17].

#### Statistical language and packages

All statistical analysis was carried out in the R language version 3.0 (www.r-project.org), and packages such as *frma* (for barcode) and *gcrma* were from the Bioconductor project (www.bioconductor.org).

## Results and discussion

### Real data

**Figure 2:**
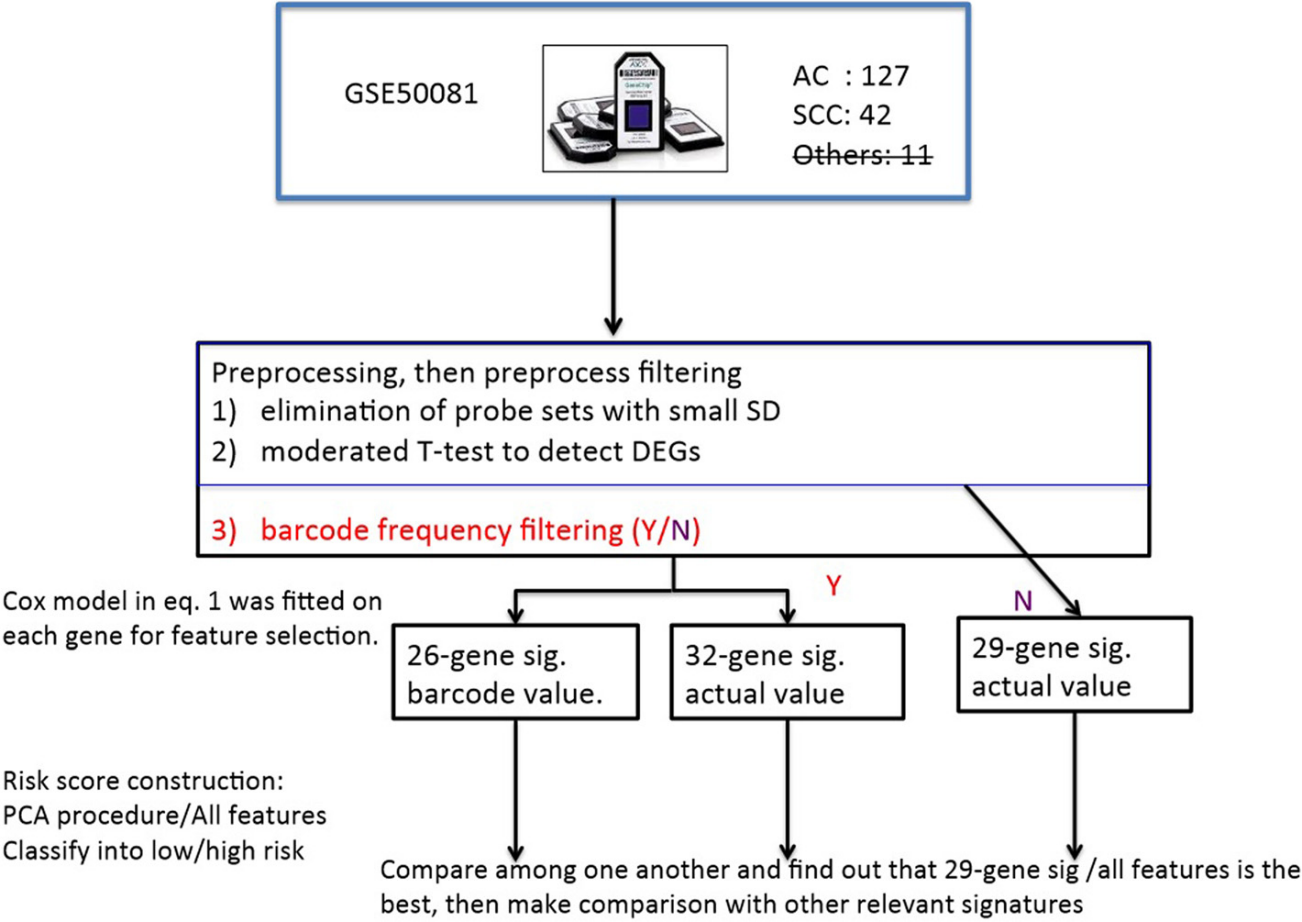
**Study schema showing how the 29-, 32-, and 26-gene signatures were obtained.** The 29-gene signature was obtained using all DEG genes; the 32-gene signature was obtained using 1889 genes with extra barcode-frequency filtering and actual expression values of these genes; and the 26-gene signature was obtained using the barcode values of those 1889 genes. We used both PCA procedure and the models with all genes as covariates to compute the risk scores and found out that even the proportion in variance of variables is higher than 95% by the selected PCs, the performance is inferior than that using all selected genes as covariates.

As mentioned in the Background section, our goal is to identify histology subtype-specific prognostic genes. Such signatures may guide the prescription of adjuvant therapies to eligible patients and avoid therapies to patients with good prognosis. To test this research hypothesis, we applied the filter feature selection method introduced in the Methods section, which is hereafter referred to as the Cox-model filter. Figure 2 highlights the study schema. As expected, the Cox-model filter identified some subtype-specific prognostic genes.

#### Feature selection using Cox models

We fit the Cox model in Equation (1) and estimated the parameters of interest for each of 6,202 genes under consideration. We examined Schoenfeld residuals for all models to test the proportional hazards assumption of a Cox model. The p-values for those tests ranged from 0.0037 to 0.9995; with 48 values being less than 0.05 and 5 less than 0.01. These numbers are much less than 5% and 1% of the total number of genes. Furthermore, there was no overlap between the genes that were identified as prognostic and those with p-values less than 0.05 in the test for proportionality. These suggest the proportional hazards assumption is plausible.

**Figure 3:**
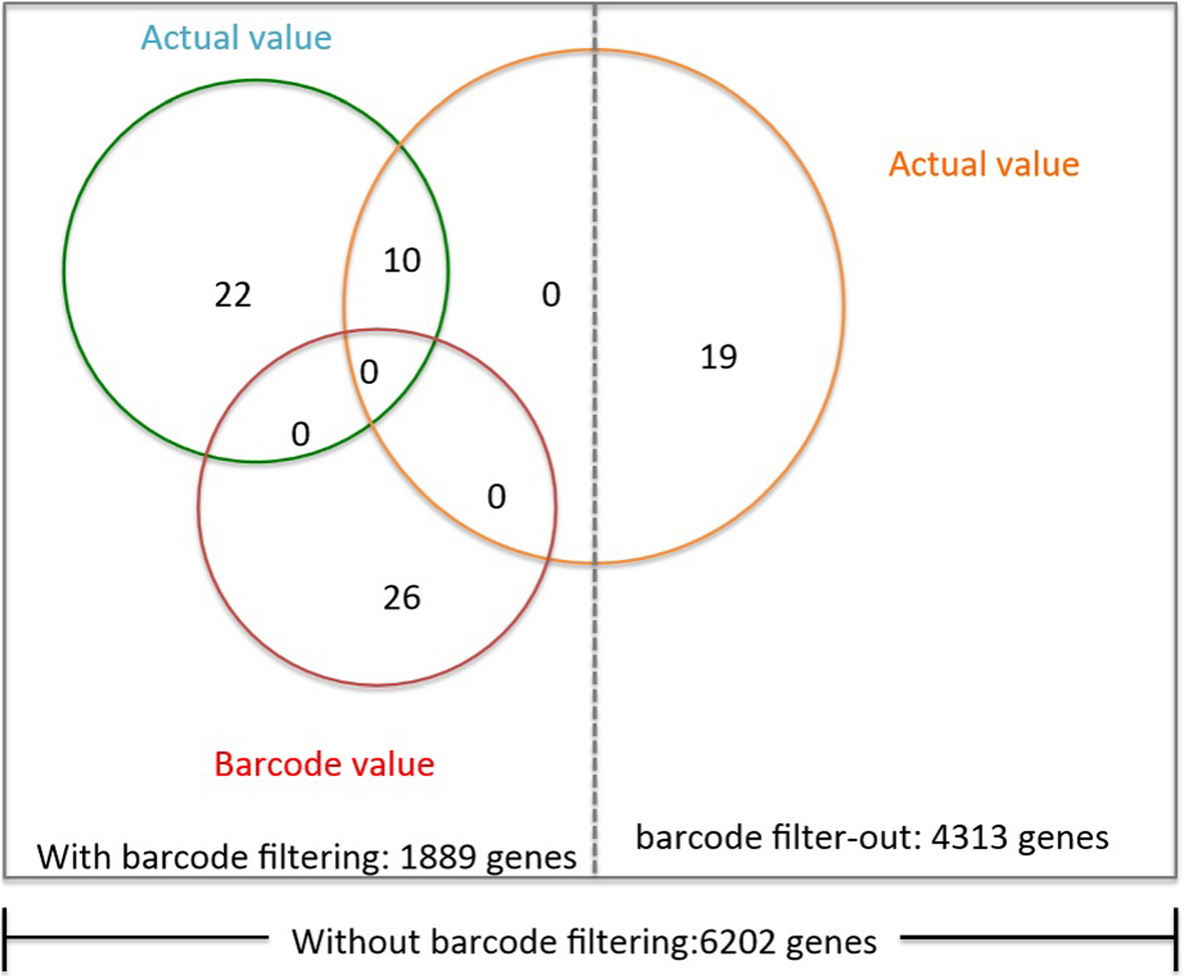
**Venn-diagram showing how the 29-, 32-, and 26-gene signatures intersect.**

Based on the actual expression values without extra barcode frequency filtering, there were 19 and 24 genes whose coefficients were statistically significantly different from zero for AC and SCC subtypes, respectively. With extra barcode frequency filtering, there were 32 genes and 12 genes with AC and SCC coefficients statistically significantly different from zero using the actual expression values while there were 26 genes and 0 gene respectively using the barcode expression values. Venn-diagrams were made to show how these three selected gene sets intersected (Figure 3).

Based on the actual expression values, we justified the existence of histology subtype-specific prognostic genes; whereas based on the barcode expression values, we identified 26 AC-specific and no SCC-specific genes. We observed, however, that the number of prognostic genes for early-stage NSCLC tends to be larger when using the actual expression values versus the barcoded values. One possible explanation was that after the actual expression values were dichotomized into barcoded values, the genes with weak or even moderate association to survival were discarded.

**Figure 4:**
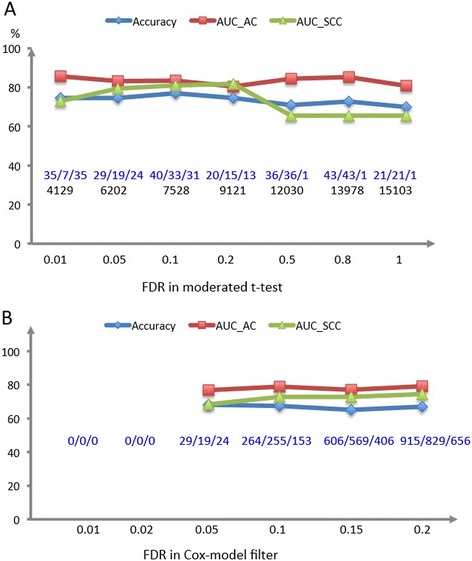
**Sensitivity curves for different cutoffs in the Cox-model filter. A)** Predictive statistics as a function of different FDR cutoff in moderated-t test filtering; **B)** Predictive statistics as a function of different FDR cutoff in Cox-model filter, using 6,202 genes. Here, No.1/No.2/No.3 represent the overall/AC/SCC size of selected gene sets, respectively; the number in the following line represents the total genes under consideration in Panel A; accuracy stands for the proportion of rightly classified patients; risk scores were constructed using PCA method since the number of selected genes is bigger than the number of samples for most values in Panel B. From these two plots, we observed that 0.05 is an optimal threshold in both steps based on the model parsimony and predictive performance.

Additionally, we conducted a sensitivity analysis to explore the effects of different cutoffs (i.e., cutoffs for moderated t-test filtering, barcode frequency filtering, and FDR of the Cox models) on the results. First, we evaluated the performance of the resulting signatures in terms of classification *accuracy* (i.e., the rate of correctly classifying samples into their survival profiles) and the area under ROC curve (AUC) statistics using different FDR cutoffs values (Figure 4) in both moderated t-test filtering and the Cox-model filter. For both steps, we identified 0.05 as an optimal value because it achieves the optimal combination of performance and model parsimony. Moreover, we found out the conclusions are consistent across different cutoffs for barcode filtering (i.e., 5% for AC and 10% for SCC, 10% for AC and 10% for SCC, 10% for AC and 20% for SCC, and 20% for AC and 20% for SCC).

#### Risk score construction

Given the number of selected genes is still large relative to the sample size, to avoid potential fitting problems arising from a regression model including all selected genes as covariates we also adopt the procedure in [19] to construct a risk score for each sample. This procedure consists of two steps. First, a principal component analysis (PCA) is conducted and the first four principal components (PCs) are recorded. Then a multiple Cox regression model is fit with the first four PCs as covariates, and the risk scores are calculated using the coefficients of these four PCs in the Cox model as weights. We modified the procedure by determining the number of PCs based on when the proportion of variance in the selected genes explained by the PCs exceeds 95%. Moreover, since PCA only applies for continuous variables, we used the actual expression values for the 26 AC-specific genes identified using barcode values to construct the risk scores.

**Figure 5:**
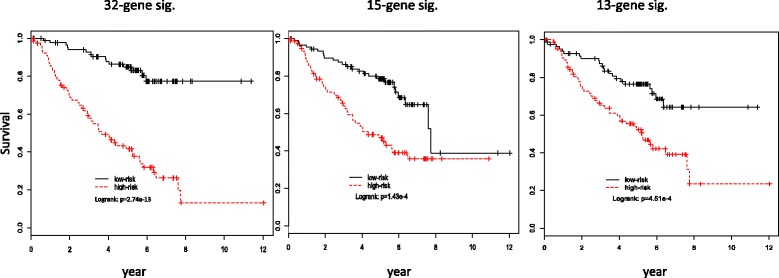
**Kaplan-Meier curves between high-risk and low-risk groups using 32-, 15-, 13-gene prognostic signatures with selected genes as covariates in risk score construction.** Patients were stratified into high-risk and low-risk groups for death on the basis of their risk scores. The cutoff was set at the mean value of those risk scores. Log-rank tests were used to compare the survival profiles of the high-risk and low-risk groups. All p-values of those tests were <0.05.

Using the mean value of those risk scores as a cutoff, we classified patients into a low-risk group or a high-risk group. Overall, the results from the PCA method is inferior to that using all genes as covariates even though the explained proportion of variances by those PCs exceeds 95%. We obtained Kaplan-Meier curves using the resulting risk scores (Figure 5), and compared the two curves using log-rank tests (Table 1).

**Table 1:** **The performance of different prognostic signatures**

**Signature (# AC/# SCC)**	**# Low /high risk**	**# of PCs/ % of variance for AC, SCC**	**Accuracy (%) All (AC/SCC)**	**AUC (%) (AC/SCC)**	**p-value (log rank)**
A: Using all covariates in the cox model for risk score construction					
29-gene (19/24)	91/78	--	72.19(73.23/69.05)	83.17/79.29	1.74 × 10^-9^
32-gene (32/12)	87/82	--	74.56(76.38/69.05)	79.82/79.57	2.74 × 10^-13^
26-gene (26/0) a^1^	83/86	--	62.72	69.96	1.22 × 10^-4^
26-gene (26/0) b^1^	88/81	--	63.31	73.90	9.01 × 10^-5^
15-gene by Zhu^2^	90/79	--	63.31	68.99	1.43 × 10^-4^
13-gene by Guo^2^	84/85	--	63.31	67.55	4.51 × 10^-4^
B: Using the modified PCA procedure for risk score construction					
29-gene (19/24)	86/83	3/96.19, 4/95.75	68.05(70.87/53.19)	76.72/68.26	3.34 × 10^-7^
32-gene (32/12)	86/83	12/95.75, 7/96.63	65.68(66.93/61.90)	77.18/71.89	2.26 × 10^-5^
26-gene (26/0) a^1^	81/88	16/95.76	60.36	66.59	1.22 × 10^-3^
15-gene^2^	91/78	3/96.41	60.36	64.04	1.34 × 10^-3^
13-gene^2^	91/78	2/96.71	60.36	58.20	1.34 × 10^-3^

Note: 29-gene signature was obtained using all 6,202 genes under consideration; 32-gene signature was obtained using 1,889 genes with extra barcode-frequency filtering and actual expression values of these genes; 26-gene signature was obtained using the barcode values of those 1,889 genes; 15-gene signature was the one in Zhu’s study [19]; 13-gene signature was the one in Guo’s study [24].

^1^Those statistics were computed for all samples pretending AC-specific genes are common prognostic genes for both AC and SCC; a: abbreviates for actual values and b: abbreviates for barcoded values. ^2^Those are AC/SCC common genes.

#### Comparison with relevant prognostic signatures

Although clinical-pathological staging is the current standard for determining NSCLC prognosis, a number of gene expression signatures have been reported to have better performance on predicting survival of NSCLC patients. Shao et al. [23] provides a comprehensive review on those prognostic gene expression signatures.

Both the 29 (19 AC/24 SCC specific)-gene and 32 (32 AC/12 SCC)-gene signatures outperformed the most relevant 15-gene signature, suggesting that using subtype-specific genes can improve the prediction of one patient’s prognostic status (Table 1). Moreover, these two signatures showed substantial superiority over the 13-gene signature proposed by Guo et al. [24]. Despite these promising results, additional evaluation of these signatures on an independent dataset is warranted since they were trained and tested on the same data set.

Unsurprisingly, there is almost no overlap of genes between our signatures and other relevant ones. In fact, inconsistency among gene expression signatures developed even on the same data is very common [25,26]. This may be due to the noisy nature of microarray data, the relatively small sample size of a microarray study, and different preprocessing and downstream statistical methods used to define a signature. It is common practice then to examine the underlying pathways of selected genes to identify possibly shared pathways working together towards a biological effect [27]. Pathway analysis using STRING [28] for the 32-gene, 15-gene and 13-gene signatures is presented in Additional file 1, and the constructed functional protein-protein networks for these three signatures using STRING are presented in Additional file 2.

#### Applying the Cox-model filter to an RNA-seq data

Because microarray technology has many weaknesses, we applied the procedure to an RNA-seq data set with 489 AC and 488 SCC samples downloaded from The Cancer Genome Atlas (https://tcga-data.nci.nih.gov/tcga/), dated August 13, 2014. Our primary objective is further testing of the existence of subtype-specific prognostic markers using data from a more advanced sequencing technology. Counts-per-million (CPM) values were calculated and log_2_ transformed by the voom function [29] in the limma package. Only those patients without adjuvant treatments and in early tumor stages (i.e., stage I and II) were included in analysis. These criteria excluded almost 85% of patients, leaving 62 SCC and 81 AC patients for the downstream analysis. Since the barcode algorithm does not apply to the RNA-seq technology, only the analysis using the actual expression values was conducted. None of the β_2_s and β_3_s in the Cox models was statistically significantly different from zero. 3,882 DEGs were identified using cutoff values of 0.05 and 2.0 for FDR and fold change, respectively. The Cox model-based filter algorithm was applied to these DEGs again, and no genes were identified as statistically significant. To assess the sensitivity of the FDR threshold in the cox models, we also used more liberal values (0.1. 0.15, 0.2, 0.25, 0.3). However, the conclusions did not change.

More interestingly, we included normal controls in order to identify potential prognostic genes with altered expression relative to the original normal expression variability. Using those patients having paired controls, we took the difference of log_2_(CPM) values between tumor and matched normal samples for each gene. The resulting differences instead of the actual log_2_(CPM) values were used in the Cox models. Notably, we relaxed the requirement of early histology stages and adjuvant treatment naïve in this analysis because of the limited number of patients with paired controls. Based on the DEG’s and using an FDR cutoff of 0.3, there were 5 and 17 genes with β_2g_ and β_2g_ + β_3g_ being statistically significantly different from zero respectively. Based on all genes and using an FDR cutoff of 0.25, there were 33 and 79 such genes. In summary, including normal controls might be helpful for identifying the subtype-specific prognostic genes. Further investigation is warranted. Nevertheless, our focus in this article is not on such genes. Given the fact that it is not common practice in the clinical setting to have normal controls, we believe a direct comparison between SCC and AC subtypes is of practical importance.

#### Comparison with other algorithms and further validation using the RNA-seq data

Here, we selected 32-gene signature obtained using actual values of 1,889 genes as the final model. First, we evaluate the goodness-of-fit of this model. Based on the likelihood ratio test, 32(32 AC/12 SCC)-gene models were significantly better than null models (p = 0.0002/0.0369 for AC/SCC, respectively). The 32-gene signature is listed in Table 2.

**Table 2:** **32-gene signature**

**Gene**	**β**_**AC**_	**β**_**SCC**_
CCRN4L	0.1107	
MYBL2	0.2637	
FOXM1	0.1565	
WRB	-0.2001	0.8382
RCAN2	-0.2258	0.417
TMEM243	0.8313	-1.0549
GTSE1	-0.4777	
TOX	-0.5018	0.1849
RAPGEF5	0.0773	-0.4773
ABCA8	0.095	-0.2682
BMP1	0.227	
KCNS3	0.195	-0.193
FST	0.2765	
CHRDL1	-0.1046	
TPX2	0.2824	
CTSV	0.4525	
PREPL	-0.386	-0.3739
LMBRD1	0.3392	-1.1411
PID1	-0.323	-0.5716
ZBED2	0.1738	
NUSAP1	-0.0237	
GINS2	0.3008	
ALDH6A1	-0.4107	
KIF18B	-0.5523	
CERCAM	0.0772	
PDPK1	-0.746	0.9029
CMIP	0.2109	
PARM1	-0.0172	0.1751
BRD4	0.778	
CDT1	-0.2528	
SLC38A7	-0.0758	
SHE	0.3078	

Note: 32-gene signature was obtained using 1,889 genes with extra barcode-frequency filtering and actual expression values of these genes.

Second, we evaluate the adjusted prognostic capacity of this signature after controlling for other clinical pathological factors (including gender, age, stage, and smoking status). By fitting an extra Cox-regression model with the constructed risk score from the prognostic gene signature and those clinical factors as covariates for each subtype, we found that the models including extra clinical factors are not statistically better than the models without them (p = 0.3126/0.0536 for AC/SCC). We interpret this finding with caution because for the SCC subtype, the p-value is only slightly larger than 0.05.

Finally, in order to further evaluate the proposed Cox-model filter, we compared its performance with two state-of-the art algorithms, *glcoxph* and *Coxpath* [30]. Two R packages glcoxph and glmpath are used for *glcoxph* and *Coxpath* analysis, respectively. Here, we applied both algorithms separately to SCC and AC samples to identify subtype-specific genes. The results (Table 3) indicate that the Cox-model filter performs comparably with these two algorithms when either testing on microarray data itself or validating on the independent RNA-seq data.

**Table 3:** **Comparison with other algorithms**

**Algorithm**	**# of genes (AC/SCC)**	**Accuracy**	**AUC**	**p-value**
A: Comparison with other algorithms on the microarray data itself				
Cox-model filter	32(32/12)	74.56(76.38/69.05)	79.82/79.57	2.74 × 10^-13^
Coxpath separate	142(108/40)	81.66(83.47/76.19)	90.95/87.85	0
glcoxph separate	11(1/10)	66.27(60.63/83.33)	69.99/91.42	3.91 × 10^-2^
B: Further validation those signatures on the RNA-seq data				
Cox-model filter	30(30/11)^1^	56.8(52.86/61.82)	52.19/68.14	0.305
Coxpath separate	126(97/32)^1^	52(50/54.56)	47.29/67.36	0.272
glcoxph separate	9(1/8)^1^	55.20(51.43/60)	57.21/56.31	6.86 × 10^-2^

Note: In this comparison, we used 1,889 genes with extra barcode-frequency filtering and their actual expression values. ^1^There are some genes missing from the resultant signatures since the RNA-seq data is on a different platform from microarray data.

### Synthesized data

To explore the characteristics of our proposed procedure, we used the actual expression values for 1,889 genes after barcode filtering to conduct a simulation study. Specifically, we randomly selected 4 genes –CERCAM, ITGA5, MTHFD1L, and PLOD1 – to be prognostic markers. The expression values of these 4 genes were represented by X1 ~ X4, respectively.

**Extreme case 1: mutually exclusive markers for each subtype.** In this case, AC and SCC have completely distinct sets of markers. The hazard functions are specified as follows,

$$ \begin{array}{l}{\lambda}_{SCC}={\lambda}_0 exp\left(1.42{X}_1-0.75{X}_2\right)\\ {}{\lambda}_{AC}={\lambda}_0 exp\left(0.225{X}_3+0.177{X}_4\right)\end{array} $$

among the remaining genes, we randomly selected 96 in order to have a total of 100 features under consideration. Thus, all but the first four genes are random noise. The survival time for each patient was simulated via a Cox-exponential distribution [31], and the censoring rate was fixed at 30%.

**Extreme case 2: no subtype specific prognostic genes.** In this case, we assume AC and SCC share identical prognostic markers,

$$ {\lambda}_{AC\&SCC}={\lambda}_0 exp\left(1.42{X}_1-0.75{X}_2+0.75{X}_3-0.59{X}_4\right) $$

for each case, we simulated 500 datasets and applied our proposed method to them.

#### Simulation results

In Extreme case 1, β_2_s for genes 3 and 4, and (β_2_ + β_3_)s for genes 1 and 2 are expected to have non-zero coefficients. However, the AC-specific genes (i.e., genes 3 and 4) tend to be identified as the common prognostic markers for both subtypes (Table 4A). This unexpected finding could be due to the correlations among the four genes.

**Table 4:** **The simulation results**

	**Actual value (frequency %)**		**Barcoded value (frequency %)**	
	**AC (β**_**2**_ **)**	**SCC (β**_**2**_ **+ β**_**3**_ **)**	**AC**	**SCC**
A. Simulation 1: a case of mutually exclusive markers for each subtype				
Gene1	0	100	0	100
Gene2	0	100	0	52.8
Gene3	100	99.6	97.4	46.2
Gene4	90.6	100	97.8	100
Ave.# of selected genes	18.53	17.82	10.00	9.98
B. Simulation 2: a case of no subtype specific prognostic genes				
Gene1	100	100	100	99.8
Gene2	100	100	100	100
Gene3	100	100	100	100
Gene4	100	100	100	100
Ave.# of selected genes	33.57	29.88	27.28	19.26

Note: Frequency represents the percentage of being non-zeros among 500 replicates; Actual value represents the analysis conducted using the actual gene expression values; barcoded value represents the analysis conducted using the barcode expression values.

Varying the coefficients for genes 1 and 2, we found that when the signals are weak or moderate for the SCC subtype, the corresponding coefficients of (β_2_ + β_3_)s for genes 1 and 2 tend to be zero (data not shown). This implies that unless the signals are strong, as for gene 1 with a coefficient of 1.42 in the first simulation, the proposed procedure is highly likely to miss the SCC-specific prognostic markers. Again, one explanation is the correlation structure among genes.

In Extreme case 2, we found both β_2_ and β_2_ + β_3_ were non-zero for genes 1-4 in all 500 simulated data, as expected (Table 4B). Overall, when using the barcoded values, the total number of selected genes is substantially less than when using the actual values. Moreover, the false positive rate as indicated by the average number of selected genes is high. This may be due to the fact that our method cannot filter out (irrelevant) genes that are highly correlated with relevant ones. Additional care is needed to eliminate these false positives. For example, the pathway information may be used as a priori to distinguish between relevant and irrelevant markers.

To explore if the imbalance size between the two subtypes and the complicated correlation structure among genes influence the performance of the Cox-model filter, we conducted extra simulations. For those simulations, we used independent normally distributed random variables. The results, presented in Additional file 1, justify our two assertions. Specifically, if the sample size of a subtype is substantially smaller, then its corresponding subtype-specific genes are likely to be missed unless their effect is adequately large. Second, the inferiority of modeling parsimony in the Cox-model filter is due to it cannot eliminate those (irrelevant) genes highly correlated with relevant ones.

## Conclusions

Given the fundamental differences in the underlying mechanisms of tumor development, growth, and invasion between AC and SCC of NSCLC patients, we hypothesized that there exist histology subtype-specific genes relevant to prognosis of patients. As expected, some subtype-specific genes do emerge when we applied the proposed feature selection method to real-world microarray data.

Nevertheless, the study itself has several limitations. First, only a single microarray experiment was considered. Moreover, the number of AC samples is three times that of SCC samples, causing an imbalance between the two subtypes. A larger data set that integrates many relevant microarray experiments is desired because the results based on such an integrated data set might be more statistically powerful and generalizable to a broader population. Of course, the issue of batch effects when combining data from multiple microarray experiments conducted in different laboratories must be considered carefully. A highly desirable feature that such big datasets should possess is to include gene expression profiles from normal controls.

Second, microarray technology suffers from being insensitive to detecting genes with low expression values, i.e., 1 to 10 copies per cell. Thus, we applied the proposed procedure to an RNA-seq data, given that RNA-seq technology has perfect precision and sensitivity to detect low-expressed genes. However, we did not identify any prognostic signatures most likely because the study population in the RNA-seq data is not homogenous as it is with microarray data and those patients have not been followed up for an adequate period.

Third, the proposed filter feature selection method cannot evaluate additive effect of genes. Thus genes with large coordinated effect but small individual effects on prognosis are highly likely to be missed. We are currently working on a new approach that uses the corresponding partial likelihood and an embedded feature selection algorithm to simultaneously select relevant genes and estimate risk scores. Using the integrated data from multiple experiments and this embedded feature selection method, we will revisit this hypothesis of the existence of histology-specific prognostic gene signatures.

Finally, the proposed method is an individual-gene-based method, which ignores the biological information from the corresponding functional networks of those genes. Such information is useful for gleaning insight on identification of true genetic markers associated with phenotype of interest. These questions of how to further stratify based on functional relevance or to incorporate biological information as a priori information for feature selection will be important to consider in our future work.

Despite these limitations, our proposed method nevertheless has its merits. It is conceptually simple and straightforward to implement. Furthermore, it saves on computing time because it does not require optimization of tuning parameters via cross-validation. Therefore, we expect that researchers, especially those with minimal statistical knowledge and experience, can readily adapt this method in their own research settings.

## Reviewers’ comments

### Reviewer number: 1 Dr. Leonid Hanin, Idaho State University, United States of America

The article represents a hodgepodge of references to various methods and results related to the analysis of microarray gene expression data and prognostic genomic signatures of cancer. The methodology is dubious, and its relevance to the problem at hand has not been adequately justified. Statistical analysis in the article depends on numerous, and partly untestable, prior assumptions that were not even mentioned. The all-important computational details were not provided, and I have little confidence that computations were carried out correctly, whatever “correctly” means. Selection of methods, parametric families of distributions and cut-offs is arbitrary and is not accompanied by any discussion or sensitivity analysis. The exposition is extremely sloppy, and many sentences seem to be just meaningless combinations of various pieces of terminology.

In my opinion, the article represents a pointless fishing expedition with inadequate net. I have a strong feeling that the article falls into the “junk science” category, and I believe its publication would be an embarrassment to the journal.

Authors’ response: *We feel our manuscript might have been misinterpreted. In order to fix this, we have edited this manuscript sentence by sentence. Then we had a native English speaker read and edit the whole manuscript. Moreover, we have added many computational details, which we intentionally omitted from the original submission as we felt with those details, the manuscript might look more like a technological report. Obviously, we made the wrong choice. We hope these revisions have improved the clarity of our manuscript.*

Quality of written English:

Not suitable for publication unless extensively edited.

### Reviewer number: 2 Dr. Limsoon Wong, NUS, Singapore

This manuscript studies gene expression data of two lung cancer subtypes. It specifically investigates whether there are subtype-specific prognostic biomarkers. Using the methodology proposed in the paper, no significant biomarkers are found. The manuscript then concludes the absence of such biomarkers.

I think this study is deficient in the following aspects:

1/ Prognosis depends on the treatment used. If the treatment is up-stream of both the subtypes, the success of the treatment may not be subtype dependent. If the treatment is down-stream (i.e., subtype specific), its success of course depends on the subtype. A subtype can only be considered to have poor prognosis if there is no available effective treatment for it. It seems that the authors have not considered this aspect. So I think the results in the paper have not been interpreted soundly and the conclusion as made in the paper is not stated correctly.

Authors’ response: *As we mentioned in the****Background****section, for both subtypes of NSCLC patients at early stages, the surgical resection is the only effective treatment. However, even among patients having the surgery, half of them die of tumor recurrence. Although subtype-specific downstream treatments are available, their effectiveness is also very limited. Both AC and SCC patients have poor survival rates, which motivates us to identify gene expression prognostic markers for risk stratification and potentially more personalized medicine. As the tumor development mechanisms are fundamentally different between two subtypes, we hypothesized that there may exist prognostic genes that are specific to a certain subtype. We then evaluated the difference in prognostic value of a given gene for the two subtypes by testing the interaction term between gene expression and subtype in a Cox regression model. Since no significant interaction was identified, we concluded that there is no evidence to support the subtype-specific gene expression prognostic markers. We have emphasized this point specifically in the manuscript.*

2/ The authors proposed an specific methodology to identify subtype-specific prognostic genes. Even ignoring issues raised in 1/, one can only conclude (i) the inability of the proposed methodology to find such biomarkers, rather than (ii) the absence of such biomarkers. Moreover, the proposed approach is quite simple minded and, I suspect, is not much better than the common individual-gene-based approach studied in e.g., Venet et al. (PLoS Computational Biology, 7(10):e1002240, 2011). So scenario (i) is more likely the case. It does not rule out the possibility of more advanced methods, such as pathway-based analysis (e.g. Bioinformatics, 30(2):189-196, 2014), may have a better chance of finding such biomarkers.

Authors’ response: *We agree with the reviewer that we can only conclude (i) and not (ii). To clarify this point, we 1) use the simulations to show the proposed procedure can not identify the significant interaction terms (i.e., the subtype-specific genes) when the signals of such terms are weak or even moderate, and 2) present the limitations of this study in the****conclusions****section and carefully choose words like “no evidence of” rather than ” no existence or absent of such biomarkers.”*

*Our work here is one among the first efforts to explore subtype-specific prognostic genes. Our proposed method is “simple-minded”, which makes it easy for a biologist or clinician to understand and interpret. Although the method may not be statistically optimal, we hope it will spark interest in this research area, and ultimately lead to the development of more advanced methods and better understanding of the prognostic value of gene expression data for each NSCLC subtype.*

3/ I find the description of the method a little hard to comprehend in a single reading. Its presentation needs to be improved to make it more readable for a broader audience.

Authors’ response: *We have edited the description of the method extensively.*

Quality of written English:

Needs some language corrections before being published.

### Reviewer number: 3 Dr. Jun Yu, Chinese Academy of Sciences, China

Report form:

Tian et al. proposes a simple filter-based feature selection algorithm in a Cox regression model to analyze some real microarray data and found no evidence supporting the existence of histology-specific prognostic gene signature for early-stage AC and SCC samples. The authors suggest a 31-gene prognostic gene signature in addition to what were evaluated, such as 15-gene and 144-gene signatures. As it is nothing wrong to explore better algorithms for gene expression analysis, my major concerns here are several folds. First, microarray technology has is lethal weakness: poor in its dynamic range, i.e., it cannot distinguish the difference among lowly-expressed genes (in arrange of 1 to 10 copies per cell) and highly expressed genes (say a few hundred copies per cell). As a result, transcripts in a copy number range of 0.1 to 10 are not easily included in the analysis, which are most likely filtered out as noise after normalization. Second, genes are regulated at different levels so that they need to be further stratified for any in-depth analysis in terms of tissue-specificity and functional relevance (for an example, please see: Chen et al. [32]). Third, normal expression controls are of essence for the identification of gene expression differences. For instance, the altered expression of a gene in a tumor tissue is only relevant to its original normal expression variability. Furthermore, an altered expression may also be related to its variability as its normal expression may have a very broad range. I would like to see some additional analyses and discussions based on these principles.

Authors’ response: *We thank the reviewer for his fair comments. Here we address the points he raised one by one.*

*1) We think that the weakness of microarray technology in its limited dynamic range can be regarded as one limitation of this study. To address this issue, we investigated experiments conducted on RNA-seq, which has a much better dynamic range. Specifically, we downloaded RNA-seq data from the Cancer Genome Atlas (**https://tcga-data.nci.nih.gov/tcga/**) and applied our proposed procedure. We still failed to identify any subtype-specific prognostic signatures. We have added a discussion of the analysis using the RNA-seq data to the****Results****section.*

*2) Further stratification for any in-depth analysis in terms of tissue-specificity and functional relevance is an interesting topic that we would like to explore in the future. In this study, our major objective is to introduce the method/procedure we proposed to test the research hypothesis on the existence of NSCLC subtype-specific prognostic genes. It represents our first strategy to tackle this problem. More in-depth analysis will be part of future research. We have added one paragraph on the future work and indicated some improvement we can make on this method to the****Conclusion****section.*

*3) We agree with the reviewer that inclusion of normal controls might help to identify subtype-specific prognostic markers. Therefore, we did such analysis using the RNA-seq data from TCGA project. The results have been added to the****Results****section. Nevertheless, we also remark that we are more interested in a direct comparison between SCC and AC samples, aiming to explore if there are some unique prognostic markers for each subtype given that it is not common practice in the clinical setting to have normal controls.*

The tables need notes and the figure legends need to be more detailed.

Authors’ response: *Done.*

As a final note, the manuscript requires intensive editing for clarity and grammar. The authors should try to reduce the length of some long sentences so the readers would have chance to understand them in one read-through. Ten of the grammatical errors and unclear sentences/phrases are listed as an example (randomly recorded from the text; possible errors are highlighted with underscore):

1. “that a proportion of stage I subjects have poorer prognosis”
2. “identification of poor prognosis of early stage NSCLC patients will assist in the prescription and administration of additional therapeutic intervention, which (identification or intervention?) potentially leads to better survival for those patients”
3. “Microarray technology allows simultaneous monitoring and measuring of tens of thousands of gene expression”
4. “When analyzing (who?) data from a microarray experiment, a feature selection algorithm is usually considered to tackle with difficulties associated with the problem of the number of covariates being much larger compared with to the number of samples, and to define a relevant gene subset informative about the underlying differences among different phenotypes”.
5. “a feature selection algorithm can be categorized into three types”
6. “penalty instead and claim (that) the proposed method has better stability, saves computing time, and achieves the global optimum”
7. “there exists fundamental differences”
8. “In addition, the expression barcode values [15,16] instead of the actual expression values are proposed to be used, which eliminates genes with weak association”.
9. “that demonstrated a certain degree of variation across samples in each study were selected”.
10. “we hypothesized that there might exist some specific genes relevant to survival rates uniquely for each of these two histology subtypes”

Authors’ response: *Thanks for highlighting these sentences for illustration. We have made extensive edits to our writing.*

Quality of written English:

Not suitable for publication unless extensively edited.

## Round 2

### Reviewer 1

As a result of extensive editing, the exposition quality of the paper has improved substantially and its content has now become more transparent. However, I still have many reservations about the study’s set-up, methodology, and results.

1. I believe the paper confuses biological aggressiveness of the types of cancer under study and clinical prognosis. While it can be hypothesized that the former depends on genes the latter depends critically on many non-genetic variables (smoking status, family history of lung cancer, tumor grade, tumor size and localization, type of treatment etc.). Any sound genomic study should control for these important non-genetic variables.

Authors’ response: *We agree with the reviewer that clinical prognosis may depend on many non-genetic variables. Even though the study population in this microarray study is relatively homogenous (at early stages and adjuvant treatments naïve given it has been collected for validation on the 15-gene prognostic signatures), we have added one extra model using selected genes and some clinical variables as covariates to get a better control for those clinical factors.*

2. Adenocarcinoma and squamous cell carcinoma originate from very different cell types. I think before analyzing gene expression profiles of cancer cells it would be useful to study gene expressions of their normal counterparts.

Authors’ response: *As suggested by reviewer 3 in the first round of revisions, we indeed included some normal controls in our add-on RNA-seq analysis. In that analysis, we did found some potential subtype-specific prognostic genes. The details on this analysis are presented in the****Results****section.*

*Nevertheless, given the fact that it is not common practice in the clinical setting to have normal controls we still believe a direct comparison between SCC and AC subtypes is of practical importance.*

3. Gene expression signatures obtained in the work resulted from a somewhat obscure maze of statistical manipulations with ad hoc selected parameters. But what is the general definition of genetic signature the authors are looking for? What extremal properties does it have?

Authors’ response: *By identifying a genetic signature, in general, we mean to select genes whose expression values are associated with the outcome. Those selected genes, independent of clinical markers, may provide insightful information on the phenotype under consideration.*

*We are not sure about what “extremal” means. If it means “asymptotic”, a resulting signature from a feature selection algorithm should ideally be able to identify the true predictors who are associated with the outcome and exclude the irrelevant ones, and the estimators for the coefficients of those relevant predictors are consistent (they converge in probability to their true values when the sample size tends to infinite).*

4. Given that the results of the article have consistency problems both internally and when compared with other studies and that the study with synthetic data also leads to unexpected results, one may suspect that the method employed is problematic. The ultimate test for validity of any method is its predictive power. I suggest that the method be tested against real gene expression data where the presence of stable gene expression signatures was firmly established.

Authors’ response: *We agree with the reviewer that the predictive power is ultimately important and acknowledge him for this insightful comment. We reran the analysis without using 5-year censoring (the change we made to address question 8 in the following page) and compared the resulting prognostic signature with two other existing prognostic signatures and signatures constructed using novel algorithms. It turns out that our method has good predictive power based on the considered predictive statistics.*

*Previously, we considered seriously to test our method on a real data where the presence of stable gene expression signatures was firmly established when we set up the manuscript. Nevertheless, we found that it is impossible to implement empirically. There are many gene expression data on cancers, but we cannot find a cancer data where a stable prognostic signature was firmly established, let alone those diseases are less studied than cancers using gene expression profiles.*

5. Statistical methods employed depend on many assumptions. For example, for T-test to be applicable, normalized and log-transformed raw expression signals should be i.i.d. and normally distributed. Is this even remotely the case? Also, for Cox model to work, hazard functions for different patients should be proportional. Are they?

Authors’ response: *Moderated T-tests used to filter the DEGs were conducted using limma package in R Bioconductor project. The functions in Limma have considerable good toleration to mild or moderation deviation from the normality assumption and moderated t-test is the most commonly used method to identify DEGs. Also, given in both microarray and RNA-seq data, the sample sizes for both histology subtypes are big enough for CLT to be applicable, we have no worry about the validness of moderated t-tests for filtering out non-DEGs.*

*For the Cox model filter, Schoenfeld residuals for those models were assessed to ensure the proportionality assumption was met.*

6. Statistical models for data are useful only if they fit the data well enough. No evidence for the goodness-of-fit or analysis of residuals was presented.

Authors’ response: *For the final model, we have conducted goodness-of-fit test to show this model fits well enough. One sentence describing of this analysis has been added to the****Results****section.*

7. I understand what hazard function for a patient is. What is it for a gene and for a gene-patient pair?

Authors’ response: *In this article, we consider hazard function for a patient using her/his gene expression value as a covariate. First, we considered each gene individually to filter statistically significant ones. Then, we fitted a Cox model with all selected gene expression as covariates to compute the risk score for each patient.*

8. In the case of early-stage NSCLC, selection of 5 years as a cut-off for censoring seems to be a poor choice. According to SEER data, 5-year survival for NSCLC patients diagnosed in 1998-2000 was 49%, 45%, 30% and 31% for stages IA, IB, IIA and IIB, respectively. Thus, censoring at 5 years significantly reduces statistical power of the analysis.

Authors’ response: *We highly appreciate the reviewer for this insightful comment. We have reanalyzed microarray without cutting off at 5-year and found out huge difference takes place without censoring at 5 years. The whole manuscript has been redone consequently. It is because for the microarray data, about half of patients had been followed up more than 5 years, and thus censoring at 5 years does significantly reduce statistical power of the analysis.*

9.In the Barcode algorithm, why instead of using untestable distributional prior assumptions on mu_g and tau_g one cannot estimate them from the data nonparametrically?

Authors’ response: *For the barcode algorithm, there are two relevant subsequent versions. Specifically, in the first version mu_g (the first peak in empirical density curve for each gene) and tau_g (expression values to the left of this first mode were used to get the estimate) are estimated non-parametrically. Since this version requires genes to show clear separation between low and high expression values in order to classify silenced and expressed groups, they choose to use EM algorithm to estimate those parameters with those distributional prior assumptions in the second version. Thus complete expression barcode calls for all genes on the array are possible.*

*For barcode 2.0, they have extended search over the GEO and ArrayExpress repositories to include all eligible samples (e.g., for Hgu133plus2 platform there are 18,656 samples). The resulting estimates on those parameters are deposited into an R Bioconductor package. So far, barcode 2.0 has been applied to a number of real-world data and demonstrated to have considerable good performance. Even though the authors have not provided any justification on these assumptions, these are the most commonly used ones for mean and variance in Bayesian inference.*

10. The authors stated without showing data that their results are consistent for different cut-off thresholds, significance levels, etc. What are these “results”? It would be good to present, for example, the number of genes surviving filtering as a function of these tuning parameters and other sensitivity curves.

Authors’ response: *we have made figures showing how the number of genes surviving filtering and performance statistics changed as a function of tuning parameters, which was presented in Figure*4*.*

11. How was the performance of the authors’ algorithm compared to other published studies?

Authors’ response: *So far we have compared the resulting prognostic signature to other two relevant signatures, showing the performance of our signature is comparable to that of these signatures. In addition, we have added a comparison between our proposed method and two state-of-art algorithms to the manuscript.*

12. The article still has many stylistic deficiencies that are too numerous to list.

Authors’ response: *we have made more extensive editions on the manuscript, aiming to identify and thus eliminate those deficiencies as best as we can.*

Quality of written English:

Needs some language corrections before being published.

### Reviewer 2:

1/ The work presented in this paper, at best, shows the proposed method fails to find prognostic markers for certain lung carcinoma. I do not consider this to be interesting or important.

Authors’ response: *We reanalyzed the data without censoring the survival time at 5-year, as suggested by Reviewer 1, we found out the proposed method identifies some subtype-specific prognostic markers for AC and SCC lung carcinoma. The whole manuscript has been redone accordingly.*

2/ Moreover, the failure of one method (which I doubt is anywhere near the best) is far from sufficient to justify the grand title “No evidence of …”. I expect many of the best methods to be tried. This was not done. So the paper way over claims its importance and misleads by its title.

Authors’ response: *Thanks for pointing this out. We must have miscomprehended the meaning of “No evidence of…”. To fix this, we have renamed this manuscript to provide a better outline on what the focus of this manuscript is.*

*Based on the experience from svb IMPROVER challenge, Tarca et al.* [33] *concluded there is no such thing as an optimal method that is uniformly superior for all data. Applying to suitable data, a simple-headed method like t-test based filter can outperform many complicated feature selection algorithm, as shown by Haury et al.* [34]*. We are confident about the soundness of our proposed method. Especially after lifting the 5-year censoring cutoff, the proposed method identified some subtype-specific genes and those genes behold good performance.*

3/ In the authors’ response letter, it is stated that the only successful treatment is surgical resection and that this still has 50% relapse. A reasonable postulate of the relapse is that the tissue immediately surrounding the surgery site contains some “seeds” for the carcinoma. Thus the relapse would be determined by whether this surrounding tissue contains such seeds, rather than by the (gene expression) profile tissue that was resected. Then, in this case, the method and experiments proposed by this paper (for identifying prognostic markers) does not make very strong sense to me. It may be more reasonable to profile the surrounding tissue of the site instead.

Authors’ response: *The word “only” should be “most”, sorry for this mistake. Studies have recently shown that survival of early stage NSCLC patients can be improved with adjuvant chemotherapy. Therefore, researchers have began to seek genetic prognostic markers, which may be predictive of survival benefit from such therapy and thus be guide of personalized medicine to improve their survival.*

*Additionally, as we understand surgeons usually resect some adjacent normal tissues in order to eliminate those “seeds” for the carcinoma when they perform the surgery. Based on these and gene expression profile in primary tumor may be also a good indicator on how invasive tumor cells are, we believe it is reasonable to explore on the prognostic gene markers.*

4/ The abstract claims a 31-gene prognostic signature. This contradicts the conclusion that there is no prognostic signature. This is very confusing.

Authors’ response: *Sorry for the confusion. Actually there is no contradiction at all. The 31-gene prognostic signature is for the one in common for both SCC and AC subtypes. Our conclusion is that we cannot find histology subtype specific prognostic signature, which does not imply there is no common prognostic genes for those two subtypes. To eliminate the possibility of such confusion may arise again, we have revised the wording in the corresponding sentences to clarify what kind of signatures they are.*

*Notably, without cutoff at 5-year the results are totally different. But we still have explicitly differed the concepts of subtype-specific and general (common) in both subtypes.*

5/ Also, the paper appears to try to “sell” the proposed method for finding prognostic markers. If this is the purpose, the authors should compare it to other state-of-the-art methods and clearly prove its superiority in terms of generality, reproducibility, etc.

Authors’ response: *The proposed method is, to our best knowledge, one of its kinds so far. However, given we are trying to show a method specifically for finding histology-subtype unique prognostic makers indeed, we have applied some methods separately to SCC and AC samples, identified the corresponding signatures and compared their performance with that of our proposed method. The results have been added to the manuscript.*

Overall, I do not know what the focus of the paper is. If it is to show the non-existence of prognostic markers for the lung carcinomas considered, it has failed to so do convincingly. If it is to show that the proposed method is good, it has failed to demonstrate its superiority against current state of the art. If it is just to show that the proposed method cannot find any prognostic markers for the carcinomas considered, I do not see why it is interesting. I am inclined on rejecting the paper.

Authors’ response: *We hypothesize that there is separate set of prognostic genes that are different among SCC and AC lung carcinomas. To test on this research hypothesis, we propose a feature selection algorithm using the Cox-model in Equation*1*as a filter.*

*To further clarify our work, we have elucidated the objectives more explicitly in the****Introduction****section and added more analysis (including a comparison between our proposed algorithm and other two state-of-the art methods). We hope with these modifications, the manuscript becomes eye-catching.*

Quality of written English:

Needs some language corrections before being published.

### Reviewer 3:

The authors have revised the manuscript and addressed some of the issues raised by the reviewers, included some new data and performed additional analyses. Its English has improved but still have room to do better. For instance, the current title denies categorily the existence of subtype-specific prognostic signatures, and it appears unnecessary. The possibility actually still exists in the future when novel methods allow better data acquisition and analysis.

Quality of written English:

Needs some language corrections before being published

Authors’ response: *Thanks a lot for pointing this out, the title is obsolete given we have conducted extra analysis and modified throughout the whole manuscript. Therefore, we have changed the title to “Test on existence of histology subtype-specific prognostic signatures among early stage lung adenocarcinoma and squamous cell carcinoma patients using a Cox-model based filter” to outline and summarize our manuscript better.*

## Additional files

Additional file 1: **Supplementary materials.**

Additional file 2: Figure S1. Constructed functional protein-protein networks for 32-, 15-, 13-gene prognostic signatures.

## Abbreviations

NSCLC: Non-small cell lung cancer
AC: Adenocarcinoma
SCC: Squamous cell carcinoma
PCA: Principal component analysis
FDR: False discovery rate
GEO: Gene expression omnibus
DEG: Differentially expressed gene
LARS: Least angle regressions
