# Supplementary material for: Test on existence of histology subtype-specific prognostic signatures among early stage lung adenocarcinoma and squamous cell carcinoma patients using a Cox-model based filter
Source: Biol Direct. 2015 Apr 7;10:15. doi: 10.1186/s13062-015-0051-z (PMC4415297; doi:10.1186/s13062-015-0051-z)
Supplement: Supplementary file 1 — Supplementary materials. [file 13062_2015_51_MOESM1_ESM.doc]

## Additional file 1: Supplementary Materials

**Pathway analysis**

To explore the relationship of the 32-gene signature with other relevant prognostic signatures, the functional protein-protein interaction network using an online database called STRING ([http://www.string-db.org](http://www.string-db.org/)) had been conducted. The possible interacted functional proteins with those identified in three signatures were shown in Supp. Figure 1.

Using the feature of KEGG pathways enrichment analysis endowed inside STRING, those top enriched pathways for each signature are presented in Supp. Table 1. There are four enriched pathways overlapped between our 32-gene signature and the 15-gene signature. Nevertheless, laborious work is definitely warranted so that a biological meaningful and one all-fit-one prognostic signature for NSCLC can put into practice in clinical setting.

**Simulations using independent random variables**

Here, instead of using actual gene expression values to set up simulations, we simulated from independent normal-distributed random variables. Even though this simple setting cannot mimic the real-world applications well, we still may use it to explore the impact of imbalanced sample sizes and correlations among features.

We simulated 100 independent random variables X1~X100 and chose the first four as relevant ones. Here, we only considered the case of mutually exclusive markers for each subtype. In this case, two subtypes have completely distinct sets of markers. The hazard functions are specified as follows,

$$\begin{matrix} \lambda_{type1}=\lambda_{0}\exp(\beta_{1}X_{1}+\beta_{2}X_{2}) \\ \lambda_{type2}=\lambda_{0}\exp(\beta_{3}X_{3}+\beta_{4}X_{4}) \end{matrix}$$

again, all but the first four genes are random noises. The survival time for each patient was simulated via a Cox-exponential distribution and the censoring rate was fixed at 30 %. Varying the values of those four coefficients, sample sizes, and the ratio of size between two subtypes, we conducted several simulations. In each one, we simulated 500 datasets and applied our proposed method to them. The results are tabulated in Supp. Table 2. From it, we observe when the ratio of sample sizes between two types is 1:1, the chance of being selected is mainly dependent on the magnitude of a feature’s effect, especially when the total size is not very big. When such ratio is not 1:1, features from the dominant-sized type are more likely to be identified.

Supp. Table1. The enriched KEGG pathways in enhanced 32-, 15-, 13-gene signatures

| A. Enriched KEGG pathways in our 29-gene signature   |  |  | | --- | --- | | | | | |
| --- | --- | --- | --- | --- | --- | --- |
| ID | Pathway | # Genes | p-value | FDR |
| hsa03030 | DNA replication | 8 | 1.12E-14 | 2.65E-12 |
| hsa04110 | **Cell cycle** | 10 | 1.34E-13 | 1.58E-11 |
| hsa03430 | Mismatch repair | 5 | 1.59E-09 | 1.25E-07 |
| hsa03420 | Nucleotide excision repair | 5 | 5.55E-08 | 3.29E-06 |
| hsa04115 | **p53 signaling pathway** | 5 | 3.92E-07 | 1.86E-05 |
| hsa05215 | **Prostate cancer** | 5 | 1.56E-06 | 6.18E-05 |
| hsa05222 | **Small cell lung cancer** | 4 | 4.12E-05 | 1.40E-03 |
| hsa03410 | Base excision repair | 3 | 5.69E-05 | 1.69E-03 |
| hsa04114 | Oocyte meiosis | 4 | 1.01E-04 | 2.67E-03 |
| hsa05200 | Pathways in cancer | 5 | 7.61E-04 | 1.80E-02 |
| B. Enriched KEGG pathways in 15-gene signature by Der et al, 2014 | | | | |
| hsa05214 | Glioma | 4 | 5.36E-06 | 6.95E-04 |
| hsa05218 | Melanoma | 4 | 8.79E-06 | 6.95E-04 |
| hsa05220 | Chronic myeloid leukemia | 4 | 8.79E-06 | 6.95E-04 |
| hsa05215 | **Prostate cancer** | 4 | 2.22E-05 | 1.31E-03 |
| hsa05219 | Bladder cancer | 3 | 6.60E-05 | 3.13E-03 |
| hsa05223 | Non-small cell lung cancer | 3 | 1.46E-04 | 5.75E-03 |
| hsa05212 | Pancreatic cancer | 3 | 2.70E-04 | 8.78E-03 |
| hsa04115 | **p53 signaling pathway** | 3 | 2.96E-04 | 8.78E-03 |
| hsa05222 | **Small cell lung cancer** | 3 | 6.25E-04 | 1.65E-02 |
| **hsa04110** | **Cell cycle** | 3 | 1.71E-03 | 4.04E-02 |
| C. Enriched KEGG pathways in 15-gene signature by Guo et al, 2011 | | | | |
| hsa05164 | Influenza A | 6 | 8.02E-07 | 1.90E-04 |
| hsa05160 | Hepatitis C | 5 | 5.90E-06 | 5.30E-04 |
| hsa04623 | Cytosolic DNA-sensing pathway | 4 | 6.71E-06 | 5.30E-04 |
| hsa04622 | RIG-I-like receptor signaling pathway | 4 | 1.26E-05 | 7.46E-04 |
| hsa04620 | Toll-like receptor signaling pathway | 4 | 5.28E-05 | 2.50E-03 |
| hsa05162 | Measles | 4 | 1.53E-04 | 6.00E-03 |
| hsa04150 | mTOR signaling pathway | 3 | 1.77E-04 | 6.00E-03 |
| hsa04720 | Long-term potentiation | 3 | 4.00E-04 | 1.18E-02 |
| hsa05215 | **Prostate cancer** | 3 | 8.61E-04 | 2.27E-02 |
| hsa04916 | Melanogenesis | 3 | 1.18E-03 | 2.80E-02 |

Supp. Table 2. Simulation results using independent random variables

|  | Ratio 1:1 | | Ration 3:1 | | Ratio 1:1 | | Ration 3:1 | |
| --- | --- | --- | --- | --- | --- | --- | --- | --- |
|  | Type 1 | Type 2 | Type 1 | Type 2 | Type 1 | Type 2 | Type 1 | Type 2 |
| β_1_=-0.62, β_2_=0.45, β_3_=0.75, β_4_=0.59, N=400 | | | | | N=200 | | | |
| Gene1 | 97 | 2 | 99 | 1 | 56 | 0 | 97 | 0 |
| Gene2 | 67 | 1 | 95 | 0 | 22 | 0 | 56 | 0 |
| Gene3 | 0 | 100 | 0 | 78 | 0 | 84 | 0 | 26 |
| Gene4 | 1 | 90 | 0 | 51 | 0 | 53 | 0 | 4 |
| Ave.# | 1.79 | 2.07 | 2.06 | 1.46 | 0.84 | 1.48 | 1.68 | 0.40 |
| N=400, β_1_=-0.62, β_2_=0.45, β_3_=1.75, β_4_=1.59 | | | | | N=200 | | | |
| Gene1 | 82 | 1 | 100 | 0 | 27 | 1 | 83 | 0 |
| Gene2 | 40 | 0 | 87 | 1 | 11 | 0 | 38 | 1 |
| Gene3 | 0 | 100 | 0 | 100 | 0 | 100 | 0 | 81 |
| Gene4 | 1 | 100 | 0 | 100 | 0 | 96 | 0 | 65 |
| Ave.# | 1.23 | 2.30 | 1.94 | 2.63 | 0.38 | 2.17 | 1.30 | 1.98 |

Note: Ave.# stands for the average number of being selected genes in 500 replicates; N represents the total sample size; ratio 1:1 means the ratio between sample size in type 1 over that in type 2.

Supp. Figure 1. The functional protein-protein networks for 32-, 15-, 13-gene signatures
