# Supplementary figures and images for: Test on existence of histology subtype-specific prognostic signatures among early stage lung adenocarcinoma and squamous cell carcinoma patients using a Cox-model based filter
Source: Biol Direct. 2015 Apr 7;10:15. doi: 10.1186/s13062-015-0051-z (PMC4415297; doi:10.1186/s13062-015-0051-z)

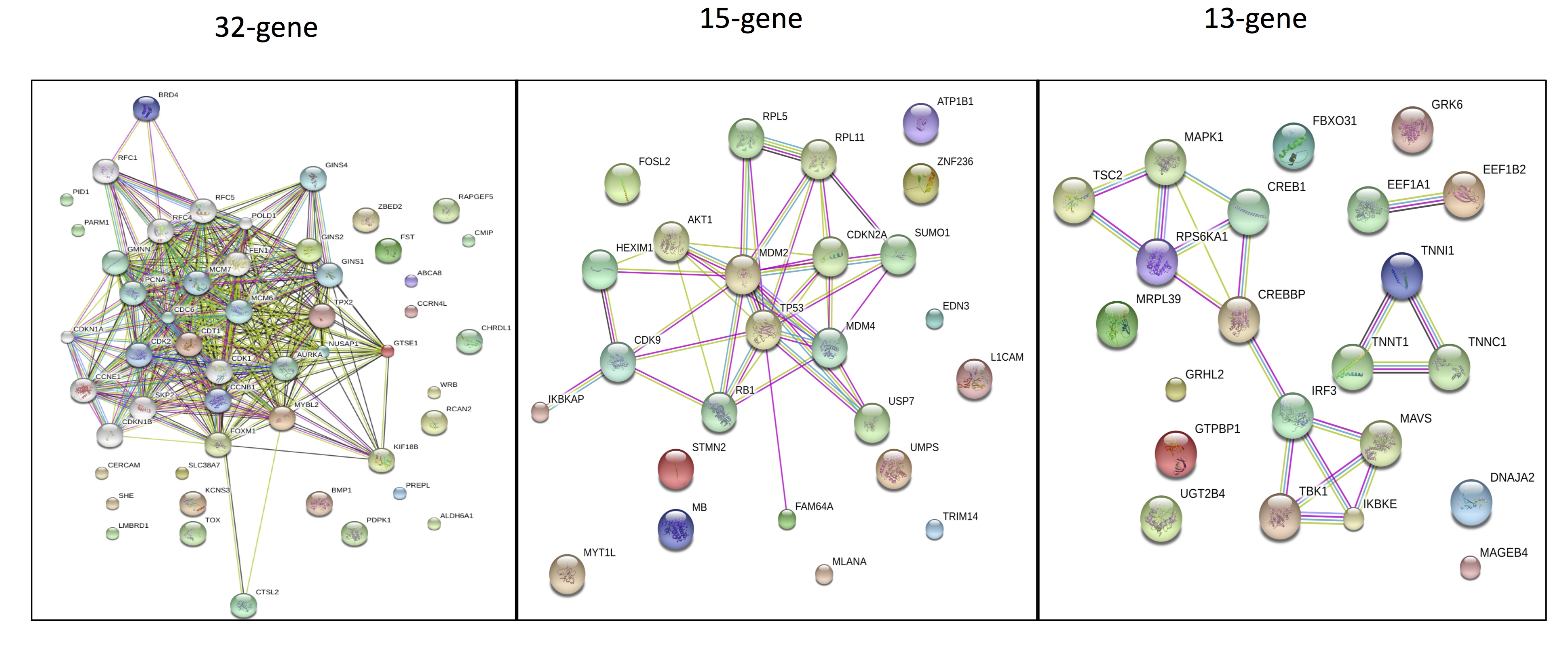

Supplement: Supplementary file 2 — Constructed functional protein-protein networks for 32-, 15-, 13-gene prognostic signatures. [file 13062_2015_51_MOESM2_ESM.jpeg]
